# Incidence of Non-Immunological Defenses of Soil White Grubs on Parasitism Success of *Mallophora ruficauda* Larva (Diptera: Asilidae)

**DOI:** 10.3390/insects3030692

**Published:** 2012-07-26

**Authors:** Marcela K. Castelo, José E. Crespo

**Affiliations:** Group of Investigation in Ecophysiology of Parasitoids (GIEP), CONICET-IEGEBA, Department of Ecology, Genetics and Evolution, Faculty of Exact and Natural Sciences, University of Buenos Aires, University City, Building II, 4th floor, lab. 99, Buenos Aires C1428EHA, Argentina; E-Mail: mcastelo@ege.fcen.uba.ar

**Keywords:** Scarabaeidae, host vulnerability, parasitoids

## Abstract

White grubs are larvae of Coleoptera of the family Scarabaeidae. They are known because of their intensive feeding habits on crop roots. *Mallophora ruficauda *(Diptera: Asilidae) is a dipteran parasitoid whose larva is a natural enemy for white grubs. This species is a solitary ectoparasitoid, where both female and larva realize different steps in the host location process. Female place its eggs in high grasslands and then, the larva finds and parasitizes the host in the ground. There are nine potential hosts in the area of action of this parasitoid; however a high preference for *Cyclocephala signaticollis *has been observed (87% of field parasitism). It is known that many insects have developed defensive and immunological mechanisms when attacked by a parasitoid, which can be behavioral, physiological, chemical or genetic. The objectives of this work were to investigate what kind of defense and non-immunological associated mechanisms the white grubs have against this parasitoid and to understand why *M. ruficauda *have such a high preference for masked chafer grubs or *Cyclocephala* species*.* In particular, for each white grub species, we asked: (1) If there is a differential behavioral reaction when a parasitoid attack is simulated; (2) If body attributes of white grubs species have influence on defense behavior, and particularly for the masked chafer *C. signaticollis*; and (3) Why this species is the most selected by *M. ruficauda*. It was found that behavioral defenses of white grubs would explain the parasitism pattern of *M. ruficauda *larvae and its preference for *C. signaticollis*.

## 1. Introduction

Parasitoids are extremely abundant insects composing, together with their phytophagous hosts and the plants they feed on, a huge percentage of the worldwide biodiversity [1]. These organisms show a wide array of lifestyles, behaviors and strategies for parasitizing and avoiding host’s defenses against them [2]. Regarding the mechanisms and parasitism strategies, Godfray (1994) [3] made an excellent contribution in compiling the most relevant information. However, host’s defenses against parasitoids have been less studied. Phytophagous insects have evolved defenses against predators and parasitoids, including chemical, behavioral, morphological, and physiological mechanisms, or a combination of each [4]. These defenses comprise steps that are hierarchical and successive. The first kind are the behavioral and morphological defenses against a direct contact of the predator/parasitoid by prevention of being discovered or resisting the initial attack (concealment, using shelters, spreading the frass, moving between feeding sites, feeding in groups, differences in coloration, reduction of sounds). Once the insect has been contacted, the next step provides protection, through hairs, spines, regurgitation, secretions, thrashing, dropping from plants, having an enlarged cuticle and by body movements against the attacker. Finally, physiological defenses act inside the prey body. These kinds of defenses include cellular and humoral mechanisms for resisting endoparasitoids, parasites, and pathogens, such as encapsulation and melanization [4,5]. Particularly, immune response is one of the most important defenses against immature wasps and flies (parasitoids) [6]. Both avoidance and physiological mechanisms have been shown to be involved in shaping the interaction of host-parasitoid systems [4]. However, there are defenses that are not considered as immunological defenses (behavioral avoidance, self-medication, behavioral thermoregulation), *i.e.*, non-immunological defenses, that have previously been disregarded, but today are considered to be frequent and relevant [7].

Defining immunity is often linked to immune system-based defenses where molecular mechanisms that recognize the invader produce responses as encapsulation, melanization or signaling cascades [7]. However, as explained by Parker *et al.* [7], in order to gain a complete picture of immunity in a particular system, holistic approaches should be undertaken. Recently it has been found that both non‑immunological and immunological defenses interact together under an environmental context showing a more complete picture of an organism’s defense against parasites [7]. Actually, studies regarding the importance of non-immunological defenses against parasites are untangling different aspects of host-parasitoid interactions which have until now been unexplained. In this work we show some non‑immunological defenses of Scarabaeidae larvae to a dipteran parasitoid. Particularly, we focus on different behaviors rhizophagous scarab larvae show that can explain the parasitism pattern of the parasitoid *Mallophora ruficauda* in nature.

One way to understand hosts behavior is by indirect observations of their parasitoids’ behavior. In this sense, many strategies for correctly assessing and determining host quality by the parasitoids might help understand the defensive responses of hosts. If different host species are incorrectly assessed regarding their quality, this might reflect that the parasitoid is also unable to correctly assess them, or that host defenses are good enough to confound the parasitoid. Some of the most studied behaviors involved in assessing host quality are the discrimination of the identity and parasitism status. As for the identity of hosts, it is well known that volatile and cuticular chemicals are involved [8,9,10,11]. Regarding the parasitism status, many examples show that parasitoids are capable of host discrimination [3,12,13,14,15,16]. Host discrimination has been defined as the ability of female parasitoids to distinguish parasitized from unparasitized hosts [17]. Since parasitized hosts often offer a lower fitness value than unparasitized hosts, it has been proposed that host discrimination should be under selective pressure since females that can discriminate hosts can avoid wasting eggs and time [15,18,19]. Inter- and intraspecific host discrimination have been described as the two modalities of host discrimination [14].

Conversely, host size is often an indicator of host quality. Under an evolutionary framework, it is thought that heavier hosts should provide better resources for parasitoids and so, they should be more parasitized [18]. Alternatively, heavier hosts might have better immune or non-immune defenses to fight off parasitoids resulting in lower parasitism [18]. There has been much elaboration on the oviposition strategies and clutch size of parasitoids and many models have shown interesting mechanisms and predictions [18]. However, studies regarding the cues used in the assessment of host size have been reduced to some hymenopteran parasitoids [18,20,21,22,23]. For instance, it has been found that after encountering a host, *Trichogramma *Westwood walks over the surface of the host and drums continually with its antennae, thereby measuring the host size [21,22,24].

Immunity and immune related responses have profound impact on performance of individuals and interactions between species and is likely to be directly involved in determining an organism’s fitness [25]. Although it would be expected that Natural Selection had shaped immune traits to optimize them, there exists great genetic variation in natural animal and plant populations probably because of environmental heterogeneity [25]. Results of integration of the consequences of resource investment to immunity as a defense under an ecological context, are crucial for untangling its implication on organisms. At a population level, some works suggest the existence of trade-offs between energy expenditure on immunity and ecological interactions such as predation, showing context-dependent survival costs to immune activation. Some of these costs might be expressed through behavioral changes, such as an augmented susceptibility to predation. For example, immune-challenged insects show significantly lower survival than healthy insects and spend more time outside burrows reacting slower to a simulated predator attack [26]. 

For parasitoids and predators, there are certain constraints regarding the kind of interactions both encounter with hosts. Hosts have different ecological and evolutionary responses to parasitoids and predators. Parasitoids complete their development feeding on a sole host, whereas most predators prey on many hosts throughout their lifetime [18]. Given the specific nature of the interaction parasitoids establish with their hosts, defenses which are most effective against many predators, may be less effective against wasps and flies parasitoids. It is known that the immune response is one of the most effective defenses hosts have against parasitism from hymenopteran and dipteran parasitoids. Insects with a strong immune response show lower rates of parasitism. 

Hymenopteran and dipteran parasitoids show important differences in the kind of interactions the parasitoid larvae and hosts have. Within Hymenoptera, these interactions are mainly physiological, immunological and biochemical processes [27]. Since early larval instars of most hymenoptera parasitoids occur in the hemocoel, encapsulation is prevented by venom and associated constituents released during oviposition [27,28]. For example, there are immunoevasive ovarial proteins coating the eggs of some braconids preventing the encapsulation [28]. Also, larval endoparasitoids have passive strategies such as oviposition in host tissues inaccessible to host hemocytes [28]. Conversely, dipteran parasitoids do not inject venom into host during oviposition and have developed two strategies to counteract the host’s immune response [27]. In Bombyliidae, Cryptochatidae, Acrocideae, Nemestrinidae, some Calliphoridae and most Tachinidae, larvae build respiratory funnels derived from host defensive cells [27,29]. Also, dipteran parasitoids may develop on host tissues that are not susceptible to host immune attack [27]. So, these kinds of parasitoids are very efficient in locating, attacking hosts and capitalizing on the host immune response [29].

*Mallophora ruficauda* Wiedemann is a pestiferous robber fly, common to the open grasslands of the Pampas Region in Argentina and is endemic to South America. Also, this species is an ectoparasitoid of Scarabaeidae larvae. In this species, immature and mature individuals use different food resources. Larvae attack and adult parasite white grubs prey mainly on foraging honey-bees and other hymenopterans. Adults are active during the austral summer (December to March) as long as there is warm and sunny weather. During their life span, mated females place eggs in clusters of 328 eggs on average on elevated sites in grasslands near bee hives [30,31]. Oviposition takes place on tall fresh or dry grasses (mainly *Carduus acanthoides* L., Asteraceae) or wire fences that serve as supports for egg clusters [30,32]. Castelo *et al.* (2006) studied the oviposition preference of *M. ruficauda *females and established that oviposition height selection contributes to larval dispersal. Particularly, monoparasitism success is maximal when egg-clutches are placed on substrates between 1.25 and 1.50 m height [31]. After seven days of sun incubation, the egg hatches, and the tiny parasitoid larvae (1.35 mm length, 0.32 mm wide) drop to the soil dispersed by the wind [33]. Once on the soil, they rapidly bury themselves and search for the host’s microhabitat detecting chemicals from its posterior intestine [34,35,36,37]. Previous studies show that the first two larval instars are capable of surviving 39 days in average without association to the host [38]. The first instar larva buries itself into the soil. After seven days in average, molting to the second instar occurs. During this instar, the host is located and parasitized. The successive larval instars are not free living and are less active. It has also been established that there is a high parasitism preference for *Cyclocephala signaticollis *(Coleoptera: Scarabaeidae) because most of the parasitism occurs on this species independently of its availability in the field [32,35]. Furthermore, in laboratory experiments it has been shown that parasitoid larvae orientate mainly to volatile chemicals from the fermentation chamber of third instar *C. signaticollis *[36,37]. Also, orientation to odors from second instar *C. signaticollis *has been observed, but this response is not so intense [39].

After parasitism occurs, *M. ruficauda *larvae remain attached to its host during the autumn and winter seasons in a low-activity state. Once the cold season is over, the host’s metabolism speeds up and it is then that the parasitoid develops quickly reaching the fifth instar in less than a month [31,32,38]. Finally, the fifth instar pupates and after 24 days the adult emerges [38]. It is noteworthy that only 15% of the first instar larvae reach the adult stage in the laboratory. The life cycle of the parasitoid is completed approximately six months after birth under laboratory conditions [38,40]. This parasitoid has a solitary lifestyle meaning that although more than one parasitoid may attach to the host (superparasitism), only one can survive and reach the adult stage. Although it has been found that *M. ruficauda *larvae are capable of host discrimination regarding its parasitism status, superparasitism is common in the field [13].

Host larvae, commonly named white grubs, belong to the Scarabaeidae family and are well known for their rhizophagous feeding habits, eating soil roots during autumn and winter [41,42,43,44,45,46]. These insects are considered serious crop pests in several parts of the world because they can incur severe losses to agriculture and damage recreational places. It has been observed that white grubs perform horizontal and vertical movements inside the soil, searching for new food or in response to temperature changes during autumn-winter [47]. They belong to four subfamilies: Dynastinae, Cetoninae, Rutelinae and Melolonthinae, which include all the phytophagous species of the family. There are nine rhizophagous species described in the Pampas Region: *C. signaticollis* Burmeister (CS), *C. modesta* Burmeister (CM), *C. putrida* Burmeister (CP), *Diloboderus abderus* Sturm (DA), *Philochloenia bonariensis* Bruch (PB), *Archophileurus vervex* Burmeister (AV), *Anomala testaceipennis* Blanchard (AT), *Bothynus striatellus* Fairmaire (BS), and *Heterogeniates bonariensis* Ohaus (HB) [47]. According to the life cycles of these species, it is known that CS, CM, CP, DA, AT, BS and HB are univoltines, whereas PB and AV would be multivoltines [47]. It was reported that *Cyclocephala* species display quiescence during the third stage, a long period where the insect shows no mobility and low metabolism [47]. This physiological reaction was observed also for other Dynastinae and Melolonthinae species, but it was not reported for Rutelinae [48,49]. However, Scarabaeidae larvae do not lose their reaction capability to changes in their physical environment during this period [49]. Castelo (2003) [33] carried out a study on the parasitism preference of this parasitoid covering the nine white grub species. That study showed that *M. ruficauda *has a high preference for CS, *ca.* 86% of field parasitism, particularly of third instar larvae. Also, it was found that the other species of the genus *Cyclocephala *are more parasitized than the other species.

Third instar larvae of Scarabaeidae species have different body sizes and activity levels. Usually, in these species, females lay eggs in groups or individually but close to each other, resulting in a gregarious habit of larvae [32,47]. Regarding the morphology, they are very similar, but there are some differences mainly in the amount and density of body setae, spines and hairs. These structures are involved in body protection against substrate and in the detection of mechanical stimuli, and can be used to sweep soil [50]. In some species, cuticular hairs reduce water loss by preventing contact between the larva and the cell wall they construct prior to pupation [51]. Alternatively, these differences could be detected by the parasitoids as specific species characteristics that could indicate the identity of prior to parasitism hosts defense capabilities as some of their non-immunological defenses. Some authors studied the behavioral defenses of white grubs and found that some species react to mechanical disturbances from natural enemies or simulated by the observer [52,53]. Also, it was found that these reactions could be age and species-dependent.

The objectives of this work were to investigate which kind of non-immunological defenses and associated mechanisms the white grubs have against this parasitoid and to deduce why *M. ruficauda *have such a high preference for masked chafer grubs or *Cyclocephala *species. Particularly, we asked the following concerning each white grub species: (1) If a differential behavioral reaction exists when a parasitoid attack is simulated; (2) If body attributes of white grubs species influence defense behavior, and particularly with regard to the masked chafer *C. signaticollis*; (3) Why this species is the most selected by *M. ruficauda*. 

## 2. Experimental Section

### 2.1. Insects

*Mallophora ruficauda *eggs were collected from herbaceous vegetation in grasslands located in Moreno (34°46′S, 58°93′W), Pilar (34°28′S, 58°55′W) and Mercedes (34°65′S, 59°43′W), three localities associated with apiaries in Buenos Aires province, Argentina, during the summers (January to March) from 2006 to 2012. We collected the egg-clutches by cutting the branch where they were attached to and stored them individually in Falcon type tubes of 30 mL until eggs started hatching. Immediately after egg hatching, neonate larvae were separated individually in Eppendorf type tubes of 1.5 mL with a moistened piece of filter paper as substrate. Drops of clean water were added when necessary to keep the humidity inside the tubes at 100%, in order to avoid dehydration of the larvae. Tubes were stored in complete darkness at room temperature between 24°C–27 °C until larvae were used in the experiments.

Hosts were collected as third instar larvae from soil samples in the same localities from where parasitoid egg-clutches were collected. The white grubs were collected from the late summer and through the whole autumn season (February to June) from 2006 to 2012 in grasslands near bee hives where robber fly activity had been observed during the previous summers. We performed a random sampling method and collected all scarab beetle larvae from each site digging the soil with a shovel at 0.30 m depth. Each individual was identified to the species level in the laboratory using the taxonomic key designed specifically to determine rhizophagous white grubs from Buenos Aires province [47]. We also recorded with a magnifier lens (16×) the number of larvae of *M. ruficauda* per beetle larva, which were attached externally to the host cuticle. Hosts were then classified according to the degree of parasitism in unparasitized and parasitized hosts. The unparasitized hosts were used for obtaining artificial parasitism while already parasitized hosts were used only to study the field parasitism pattern (Section 2.2.1). Hosts were kept individually at room temperature in black tubes filled with soil, and fed weekly with fresh pieces of carrots.

### 2.2. Parasitism Patterns

#### 2.2.1. Field Parasitism Pattern

Castelo and Corley (2010) [35] performed an extensive field study, where abundance of white grubs was studied for 4 years (1997–2000) in different locations of the Pampas Region of Argentina. Based on this information, we used for our work the more abundant species found on that study (CS, CM, CP, AT, DA and PB). Table 1 shows the percentage of parasitized white grubs found by Castelo and Corley (2010) [35]. These values reflect the percentage of white grubs for each species that had been parasitized and did not escape from parasitoid larval attack. Since this study was made in the field, it shows the parasitism pattern that results from both immunological and no-immunological defenses, and other ecological processes not controlled.

#### 2.2.2. Laboratory Parasitism Pattern

In order to artificially obtain parasitized hosts, we placed one parasitoid second instar larva on the abdomen of an unparasitized white grub with a small paintbrush and after three days, we checked if parasitism had occurred. With this procedure we could obtain parasitized hosts and also study the host preference of *M. ruficauda* larvae.

### 2.3. Parasitism Behavioral Escape

We studied if the different attachment percentage of parasitoids to the hosts was due to behavioral non-immunological defenses through aggressive reaction when the larvae touched the host. For this, we measured the reaction to a simulated parasitism and constructed a Simulated Attack Reaction Index (SARI). To simulate the parasitoid attack, we placed the white grub on a flat surface caring that the individual had not been disturbed previously, and gently punctured its dorsal abdomen just behind the third pair of legs with a blunt end stick. This procedure avoids the permanent injury of the host and the subsequent death. Then, the type and intensity of the response was registered. The type of the response was ranked according to the movement the white grub made during the procedure. If there was no apparent response, a 1 was assigned. Then, increasing values were assigned when: (2) movement of the tip of the abdomen to the head, (3) the head was moved forwards and backwards, (4) an inversion of the body loosing the characteristic “C” shape, (5) torsion of the body accompanied by an upward movement of the legs and (6) direct attack to the stick with the mandibles and legs. Also, the intensity of the response was gradually ranked between 1 when no reaction was observed, 2 when there was little movement, 3 when the insect moved very perceptibly and 4 when the individual moved violently. With this, the SARI index was constructed combining the type and intensity reaction with the following formula:

SARI = ((T/6) + (I/4))/2

where *T* stands for type of reaction and *I* for intensity of reaction. The index varies from 0 when there is no reaction and 1 when there is a violent reaction to the simulated attack. All the assays were performed under gloom light conditions and laboratory temperature (T° = 20.5–26.8 °C). Although white grubs inside the soil matrix could move more less than in a flat surface, this experimental design is able to measure a behavioral reaction reflecting an ecological relevant defense against parasitoid larvae. It is possible that host reaction could be increased by this method, because white grubs can move freely if not surrounded by soil, but since all individuals of each species were treated identically the index is affected in the same way. 

After obtaining the pattern constructed with the SARI, we analyzed the influence of host weight on host acceptance to study if there is a relation between the defensive behavior and body size. If any size dependent defense of the hosts exists, it should be evinced after weighting. For this, we weighted every host with a commercial precision scale and we compared that weight pattern and the SARI indices to the field parasitism patterns.

### 2.4. Statistics

The influence of non-immunological defenses, morphological and life cycle characteristics on parasitism by *M. ruficauda *were studied by generalized additive models with a Gaussian distribution and identity link function [54]. The smooth function was represented using thin plates regression splines with the best *k* for each variable [54]. Response variable was constructed as:

*Magnitude of non-immunological response = 100-LP*

where *LP* stands for the percentage of laboratory artificial parasitism for each species studied. Performance in SARI, individual weight and voltinism were included as explanatory variables in the models. Criterion as to if an explanatory variable was statistically significant, was evaluated with the deviance change of the model when excluded, and a posterior ANOVA between both models [55]. Also, the generalized cross-validation (GCV) score was used to further support the decision as to which model was better [55,56]. The GCV is an information criterion that is lower for better models [56]. All the analyses were done using the mgcv package available in R language.

## 3. Results and Discussion

In this work we studied the non-immunological defenses white grubs have against the parasitoid *M. ruficauda*. We found that the behavioral defenses hosts exhibit are the main factors involved to avoid the relationship in this system.

### 3.1. Field and Laboratory Parasitism Patterns

We compared the field and laboratory parasitism and found that the percentage of field parasitism (FP) is different from that of laboratory parasitism (LP), the latter being always higher for all species (Table 1). This difference indicates that the first defenses, or pre-parasitism defenses, occurring when the parasitoid contacts the host are not the only ones involved in the interaction between *M. ruficauda *and hosts. Also, the results denote that white grubs might have both immunological and non‑immunological defenses but they seem to have different relevance in each species, as is indicated by the magnitude of the difference in 100-LP percentages. The FP pattern shows the picture after host location and acceptance occurs, hence, after parasitism is established, whereas LP shows acceptance and parasitism prior to the expression of immunological defenses. It has been previously observed in the laboratory that parasitized hosts can escape from parasitism some weeks after the attack of *M. ruficauda* larvae [57], indicating that some physiological defenses could be expressed. However, to determine if multiple defenses are involved in these processes, further experimental investigation is required. Also, due to the magnitude of the differences between FP and LP, the possibility that differences in parasitism rates could arise from manipulation during experimental infection could not be discarded, as observed in other systems [58]. The most important fact is that acceptance frequencies showed the same pattern in both situations, and so could be taken as reliable indicators of parasitoid host use, which is determined by the physiological capacity of larval parasitoid to exploit the host, the behavioral ability of females to find resources, and the effectiveness of behavioral defenses of the host [58].

### 3.2. Magnitude of Non-Immunological Response

To assess the importance of non-immunological defenses on parasitism avoidance, we studied the influence of host mean weight and SARI on host escape from parasitoid contact constructing three models. In the full model, we included all the variables as explanatory variables in the model (mean host weight and SARI; model 1). Then, we deleted SARI and left host weight (model 2), and finally we made a final model which included SARI alone (model 3). The results of each model performance are shown in Table 2. 

**Figure 1:**
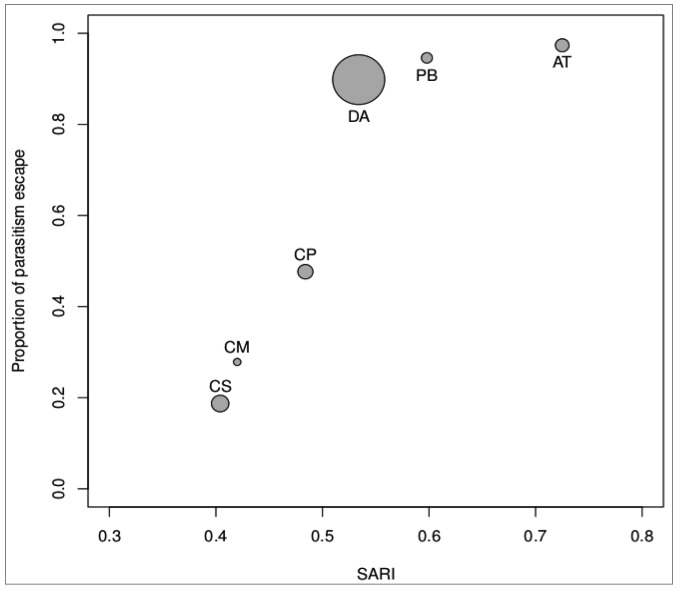
Proportion of parasitism escape of each white grub species according to the mean simulated attack reaction index (SARI). Each point shows the relative mean weight and the species name. CS: *Cyclocephala signaticollis*, CM: *C. modesta*, CP: *C. putrida*, DA: *D. abderus*, PB: *P. bonariensis*, AT: *A. testaceipennis*.

We found no initial support to keep mean host weight in the final model (*p *= 0.292, Table 2). Moreover when we removed mean host weight from the model, the deletion of that variable did not cause a significant change in deviance (ANOVA (model 1 *vs.* model 3), deviance = 0.013, residuals df = 3.077, *p* = 0.289, Table 2). When analyzing the performance of model 2, we found that the deletion of the variable SARI increased the deviance significantly (ANOVA (model 2 *vs.* model 3), deviance = 0.535, residuals df = 3.077, *p* > 0.050, Table 2). Moreover, the GCV score showed that model 1 did better than model 2 (Table 2). Then, we analyzed model 3 and found that the deviance explained is almost the same (96% *vs.* 98%, Table 2) as that of model 1. However, GCV score showed a slightly better performance of model 3 over model 1 (Table 2). So, the best model for our data included only SARI (model 3) as the variable that explained most of the variance in the model. The results of model 3 are shown in Figure 1. It shows that when the SARI increases, the percentage of escape from parasitism also increases at almost a linear rate. However, there seems to be a SARI value (~0.55) beyond which there is no substantial increase in the percentage of escape from parasitism and the relation is almost constant. This result shows that violent body movements and torsion, as a behavioral reaction to an attack, are very effective non-immunological defenses preventing a host from being parasitized.

**Table 1:** Natural and artificial white grub parasitism in the Pampas Region of Argentina. The number of larvae found in each species is indicated as: total individuals found (number of parasitized individuals), percentage of parasitism, mean body weight and the mean score in the simulated attack reaction index (SARI). *Data taken from Castelo and Corley 2010 [35]. Species: see text for details.

Species	N Field*	FP (%)*	N Laboratory	LP (%)	100-LP (%)	Mean Weight (gr)	Mean SARI score
CS	945 (180)	19.05	331 (269)	81.27	18.73	0.745	0.404
CM	182 (14)	7.69	79 (57)	72.15	27.85	0.320	0.420
CP	81 (3)	3.70	107 (56)	52.34	47.66	0.649	0.484
AT	125 (1)	0.80	76 (2)	2.63	97.37	0.586	0.725
DA	93 (2)	2.15	59 (6)	10.17	89.83	2.225	0.534
PB	1005 (7)	0.70	130 (7)	5.38	94.62	0.469	0.598
AV	18 (1)	5.56	-	-	-	-	-
BS	2 (0)	0	-	-	-	-	-
HB	3 (0)	0	-	-	-	-	-

**Table 2:** Models created to explain the parasitism patterns of the white grubs in the Pampas Region of Argentina. Mean performance in the SARI and mean weight for each species as included in every model. In each case the parameters for every model with its significance is shown. Note: EDF: estimated degrees of freedom, F: ANOVA F value, p: significance, E: estimated value for the intercept, SE: Standard error of the estimate, t: value of the t-test, Dev: deviance of the data explained.

Model	s(SARI)			s(mean.weight)			Intercept				Dev. explained	R^2^ adj	Anova (model 1 *vs* model 3)			GCV score
	EDF	F	*p*	EDF	F	*p*	E	SE	*t*	*p*			Dev	F	*p*	
Model 1	1.878	47.013	0.020	1.082	2.084	0.292	0.627	0.032	19.74	<<0.005	98%	0.952	0.013	2.041	0.289	0.018
Model 2	-	-	-	1.000	0.519	0.511	0.627	0.153	4.102	<<0.05	11.5%	−0.106	0.535	70.936	0.003	0.210
Model 3	1.923	35.34	0.008	-	-	-	0.627	0.037	16.98	<<0.001	96%	0.935	-	-	-	0.016

These kinds of defense have also been found in some hymenopterans where active defensive behaviors reduce the probability of being parasitized because the parasitoid chooses other hosts or because they directly kill the infective stage [4,59,60]. Also, it has been found that the scarab *Geotrupes spiniger* Marsham (Coleoptera: Geotrupidae) larvae were sensitive to surface contact, making a coordinated attack on a source of stimulation showing defensive behavior [61]. Some studies have demonstrated that certain defenses are more efficient against hymenopteran parasitoids than against dipteran parasitoids and *vice versa* [62]. For example, Gentry and Dyer (2002) [63] found that avoidance behaviors such as thrashing, dropping, or biting were effective against female hymenopteran parasitoids, but not against dipteran parasitoids. The fact that many dipteran parasitoids use indirect methods, such as microtype eggs or planidial larvae to infect their host, buffers the female parasitoid from coming into direct contact with the insect, thus avoiding these defensive behaviors. However, after entering the host body for the melanization process, the parasitoid attack behavior has almost no influence because once the larvae/eggs are inside the host’s hemocoel, the response is universal, both for Hymenopteran and Dipteran parasitoids [6]. For example, *Cyclocephala* grubs parasitized by larvae of *Tiphia *spp. Fabricius are likely to be found deeper in the soil than nonparasitized grubs and that this phenomenon results from a behavioral change in the host, rather than physical actions by the wasp or its larva [64]. Also, it could be that the grub’s downward movement represents a defensive response to parasitism, because movement of parasitized grubs through the soil sometimes dislodges the egg or the developing ectoparasitic larva [64,65]. Nevertheless, in this work we have found that aggressive behavior against larvae of *M. ruficauda *is a very effective strategy in white grubs that contributes to avoid attachment of the ectoparasitic larvae to its cuticle. This behavioral strategy allows scarab larvae to partially escape parasitism.

As a result of the first barrier white grubs form against this parasitoid, the parasitism preference of *M. ruficauda* by *Cyclocephala* species could be determined by the level of aggression of its host. This kind of behavior has been observed in intraspecific host instar selection. For instance, the parasitoid *Lipolexis oregmae* Gahan (Hymenoptera: Aphidiidae) can oviposit and develop on every larval instar of the host *Toxoptera citricida* Kirkaldy (Hymenoptera: Aphidiidae) but higher mortality risks exist when it develops on the fourth instar compared to the second [66]. Although there are not so many studies where interspecific host preference related to host aggressive behavioral defenses was analyzed, some authors found that sometimes more aggressive hosts might interrupt the parasitoid oviposition, thus increasing its handling time [59]. In *M. ruficauda*, the host *C. signaticollis* showed the least aggressive behavior and the highest percentage of parasitism. Moreover, the three species of the genus *Cyclocephala* show lower indices regarding aggressive behavior. Another aspect of the host's body morphology that can influence their behavioral defenses is the amount of sensorial mechanoreceptors distributed along the body of every species. It is interesting that species of the genus *Cyclocephala* have fewer setae than DA which has in turn less setae than the species AT and PB. These last two species have a high density of setae on every segment and this could provide them with a high sensibility to detect an organism that contacts it.

There are also some physical factors that can increase the host's probability of parasitism escape from parasitoids. Some studies suggest that high environmental temperatures influence the host immune system and resistance, increasing the probability of a host killing its parasitoid [67,68]. For example in some Hymenopteran parasitoids it was shown that egg hatchability, postembryonic development and successful attack are lower at high rather than at low temperatures [69,70] because of either more effective host defenses or a lower effectiveness of the substances injected by the female parasitoid at oviposition. Also, it has been observed that effective encapsulation of eggs is correlated with ambient temperature [71]. These examples suggest that an increase in the environmental temperature can increase the host defensive capabilities against immature parasitoids, resulting in a decrease in parasitization success, and can thus strongly affect the host-parasitoid population dynamics by altering the capacity of parasitoids to attack hosts [67]. According to parasitism success in *C. signaticollis*, there is some evidence that *M. ruficauda* larvae attach to preferential places in the host body and this could contribute to increase the survival probabilities of the parasitoid larva [72]. It has been shown that some immature parasitoids exploit preferentially host parts that provide privileged access to energetic resources or oxygen sources [73]. 

Although the experiments performed in this work deal with the decision of the parasitoid larva to accept, *i.e.*, decide to parasitize or not a host once it is on its body, the larva of *M. ruficauda* orientates actively to the host. In this sense, the chemical cues involved in the orientation to the host in this system are also very important. Previous studies showed that *M. ruficauda*’s larvae orientate to chemical cues from CS, CM, CP, AT, DA but not from PB. This information is relevant because it shows that non-immunological defenses are very important in most of the host species in preventing parasitism. The fact that *M. ruficauda* does not orientate to PB could be interpreted as a species with very good defenses or it could explain the phenology of this species. Phenology, *i.e.*, the timing when the vulnerable stage appears in the field, of species in a host-parasitoid system is a very important factor determining the persistence of both systems [74]. Also, spatial and temporal asynchrony could promote persistence or disruption in these systems [74,75,76]. For instance, when only a small fraction of the host population is overlapped with the parasitoid population, a partial refuge for hosts is created and persistence is increased [76]. From an evolutionary perspective, voltinism (*i.e.*, number of generations per year) can also play an important role in the host-parasitoid interactions. In this context, potential host species that reduce overlapping with the parasitoid would escape more effectively from parasitism. *Philochloenia bonariensis* is a species with a life cycle at least longer than one year of duration [47]. This characteristic could explain why this species is not parasitized. Something very similar could happen with the species *A. vervex* that has a life cycle of three years [47]. However, in the latter case, very low levels of abundance in the area of *M. ruficauda* also explains why this species is not parasitized.

## 4. Conclusions

In this work we specifically addressed the kind and influence of non-immunological defenses white grubs express in order to explain the parasitism pattern found in the field. The field parasitism pattern shows the results of many behaviors and mechanisms that lead either to parasitism or escape from it in this system, including both immunological and non-immunological defenses. We have found that the host *C. signaticollis* is the species with the lowest behavioral reaction to a simulated parasitoid attack and has the highest percentage of parasitism in the field, which could indicate that this species also has poorer immunological defenses than the other species. These results altogether reveal the main factors involved in the parasitism preference of *M. ruficauda *for species of the genus *Cyclocephala*, particularly *C. signaticollis*.

## References

[1] Hawkins B.A. (1994). Pattern & Process in Host-Parasitoid Interactions.

[2] Vet L.E.M., Hemerik L., Visser M.E., Wäckers F.E., Lewis E.E., Campbell J.F., Sukhdeo M.V.K. (2002). Flexibility in host-search and patch-use strategies in insect parasitoids. The Behavioural Ecology of Parasites.

[3] Godfray H.C.J. (1994). Parasitoids: Behavior and Evolutionary Ecology.

[4] Gross P. (1993). Insect behavioral and morphological defenses against parasitoids. Annu. Rev. Entomol..

[5] Strand M.R., Pech L.L. (1995). Immunological basis for compatibility in parasitoid-host relationships. Annu. Rev. Entomol..

[6] Smilanich A.M., Dyer L.Q., Gentry G.L. (2009). The insect immune response and other putative defenses as effective predictors of parasitism. Ecology.

[7] Parker B.J., Barribeau S.M., Laughton A.M., de Roode J.C., Gerardo N.M. (2011). Non-immunological defense in an evolutionary framework. Trends Ecol. Evol..

[8] Blomquist G.J., Bagnères A.G. (2010). Insect Hydrocarbons Biology, Biochemistry, and Chemical Ecology.

[9] Fatouros N.E., Dicke M., Mumm R., Meiners T., Hilker M. (2008). Foraging behavior of egg parasitoids exploiting chemical information. Behav. Ecol..

[10] Jones R.L., Lewis W.J., Beroza M., Bierl B., Sparks A.N. (1973). Host-seeking stimulants (kairomones) for the egg parasite, *Trichogramma evanescens*. Environ. Entomol..

[11] Morehead S.A., Feener D.H. (2000). Visual and chemical cues used in host location and acceptance by a dipteran parasitoid. J. Insect Behav..

[12] Brodeur J., Boivin G. (2004). Functional ecology of immature parasitoids. Annu. Rev. Entomol..

[13] Crespo J.E., Castelo M.K. (2009). Insights to the host discrimination and host acceptance behaviour in a parasitoid (Diptera: Asilidae): Implications for fitness. J. Insect Physiol..

[14] Mackauer M., Mackauer M., Ehler L.E., Roland J. (1990). Host discrimination and larval competition in solitary endoparasitoids. Critical Issues in Biological Control.

[15] Royer L., Fournet S., Brunel E., Boivin G. (1999). Intra- and interspecific host discrimination by host-seeking larvae of coleopteran parasitoids. Oecologia.

[16] van Baaren J., Boivin G., Nénon J.P. (1994). Intra- and interspecific host discrimination in two closely related egg parasitoids. Oecologia.

[17] Roitberg B.D., Mangel M. (1988). On the evolutionary ecology of marking pheromones. Evol. Ecol..

[18] Crespo J.E., Castelo M.K. (2008). The ontogeny of host seeking behavior in a parasitoid dipteran. J. Insect Physiol..

[19] van Baaren J., Boivin G., Nénon J.P. (1995). Intraspecific hyperparasitism in a primary hymenopteran parasitoid. Behav. Ecol. Sociobiol..

[20] Purrington F.F., Uleman J.S. (1972). Brood size of a parasitic wasp: Functional correlation with the mass of a cryptic host. Ann. Entomol. Soc. Am..

[21] Salt G. (1935). Experimental studies in insect parasitism. III. Host selection. Proc. Roy. Soc. Lond. B Biol. Sci..

[22] Salt G. (1937). The sense used by *Trichogramma* to distinguish between parasitized and unparasitized hosts. Proc. Roy. Soc. Lond. B Biol. Sci..

[23] Takagi M. (1986). The reproductive strategy of the gregarious parasitoid, *Pteromalus puparium *(Hymenoptera: Pteromalidae) 2. Host size discrimination and regulation of the number and sex ratio of progeny in a single host. Oecologia.

[24] Schimdt J.M., Smith J.J.B. (1985). Host volume measurement by the parasitoid wasp *Trichogramma minutum*: The roles of curvature and surface area. Entomol. Exp. Appl..

[25] Lazzaro B.P., Little T.J. (2009). Immunity in a variable world. Phil. Trans. Roy. Soc. Lond. B Biol. Sci..

[26] Otti O., Gantenbein-Ritter I., Jacot A., Brinkhof M.G.W. (2012). Immune response increases predation risk. Evolution.

[27] Feener D.H., Brown B.V. (1997). Diptera as parasitoids. Annu. Rev. Entomol..

[28] Pennacchio F., Strand M.R. (2006). Evolution of developmental strategies in parasitic Hymenoptera. Annu. Rev. Entomol..

[29] Stireman J.O., O’Hara J.E., Wood D.M. (2006). Behavior, ecology and evolution of tachinid parasitoids. Annu. Rev. Entomol..

[30] Castelo M.K., Corley J.C. (2004). Oviposition behavior in the robber fly *Mallophora ruficauda* (Diptera: Asilidae). Ann. Entomol. Soc. Am..

[31] Castelo M.K., Ney-Nifle N., Corley J.C., Bernstein C. (2006). Oviposition height increases parasitism success by the robber fly *Mallophora ruficauda *(Diptera: Asilidae). Behav. Ecol. Sociobiol..

[32] Copello A. (1922). Biología del moscardón cazador de abejas (*Mallophora ruficauda* Wied.). Physis.

[33] Castelo M.K. (2003). Comportamiento de localización y patrones de explotación de hospedadores (Coleoptera: Scarabaeidae) por el moscardón cazador de abejas *Mallophora ruficauda* (Diptera: Asilidae) (in Spanish). Ph.D. dissertation.

[34] Castelo M.K., Capurro A.F. (2000). Especificidad y denso-dependencia inversa en parasitoides con oviposición fuera del hospedador: el caso de *Mallophora ruficauda* (Diptera: Asilidae) en la pampa argentina. Ecología Austral.

[35] Castelo M.K., Corley J.C. (2010). Spatial density dependent parasitism and specificity in the robber fly *Mallophora ruficauda* (Diptera: Asilidae). Austral Ecol..

[36] Castelo M.K., Lazzari C.R. (2004). Host-seeking behavior in larvae of the robber fly *Mallophora ruficauda* (Diptera: Asilidae). J. Insect Physiol..

[37] Crespo J.E., Castelo M.K. (2010). Life-history traits in a parasitoid dipteran species with free-living and obligate parasitic immature stages. Physiol. Entomol..

[38] Groba H.F., Castelo M.K. (2012). Chemical interaction between a dipteran parasitoid larva and its coleopteran host: A case of exploitation of the communication system during the searching behaviour?. Bull. Entomol. Res..

[39] Crespo J.E., Castelo M.K. (2012). Orientation of M. ruficauda larvae to odors of second instar white grubs. Unpublished work.

[40] Musso J.J. (1983). Nutritive and ecological requirements of robber flies (Diptera: Brachycera: Asilidae). Entomol. Gen..

[41] Ali A.D., Harivandi M.A. (1987). White grubs in lawns. Leaflet - University of California, Cooperative Extension Service.

[42] Cherry R.H., Schueneman T.J. (1998). Insect management in sugarcane.

[43] Crutchfield B.A., Potter D.A. (1995). Damage relationships of Japanese beetle and southern masked chafer (Coleoptera: Scarabaeidae) grubs in cool-season turfgrasses. J. Econ. Entomol..

[44] Potter D.A. (1998). Destructive Turfgrass Insects: Biology, Diagnosis and Control.

[45] Salvadori J.R. (1997). Manejo de corós em cereais de inverno. EMBRAPA.

[46] Smitley D.R. (1996). Incidence of Popillia japonica (Coleoptera: Scarabaeidae) and other scarab larvae in nursery fields. Hortic. Entomol..

[47] Alvarado L. (1980). Sistemática y bionomía de los estados inmaduros de coleópteros Scarabaeidae que habitan en el suelo (in Spanish). Ph.D. dissertation.

[48] Rakesha H.S. (2007). Studies on arecanut root grub, *Leucopholis lepidophora* Blanchard and its management by entomopathogenic fungi and plant products. Master thesis.

[49] Wightman J.A. (1974). Influence of low temperature on pupation induction in *Costelytra zealandica* (Coleoptera: Scarabaeidae). New Zeal. J. Zool..

[50] Grassé P.P. (1951). Insectes supérieurs et Hémiptérodes. Traité de Zoologie. Anatomie, systématique, biologie (in French).

[51] Villani M.G., Allee L.L., Díaz A., Robbins P.S. (1999). Adaptative strategies of edaphic arthropods. Annu. Rev. Entomol..

[52] Gaugler R., Wang Y., Campbell J.F. (1994). Aggressive and evasive behaviors in Popillia japonica (Coleoptera: Scarabaeidae) larvae: Defenses against entomopathogenic nematode attack. J. Invertebr. Pathol..

[53] Villani M.G., Nyrop J.P. (1991). Age-dependent movement patterns of Japanese beetle and European chafer (Coleoptera: Scarabaeidae) grubs in soil-turfgrass microcosms. Environ. Entomol..

[54] Wood S.N. (2003). Thin plate regression splines. J. Roy. Stat. Soc. B Stat. Meth..

[55] Crawley M.J. (2007). The R Book.

[56] Wood S.N. (2006). Generalized Additive Models: An Introduction with R.

[57] Crespo J.E. (2012). CONICET, FCEyN, UBA (Buenos Aires, Argentina). Parasitism escape of white grubs. Unpublished work.

[58] Brodeur J., Geervliet J.B.F., Vet L.E.M. (1996). The role of host species, age and defensive behaviour on ovipositional decisions in a solitary specialist and gregarious generalist parasitoid (*Cotesia* species). Entomol. Exp. Appl..

[59] Firlej A., Lucas E., Coderre D., Boivin G. (2010). Impact of host behavioral defenses on parasitization efficacy of a larval and adult parasitoid. Biocontrol.

[60] Potting R.P.J., Vermeulen N.E., Conlong D.E. (1999). Active defense of herbivorous hosts against parasitism: Adult parasitoid mortality risk involved in attacking a concealed stemboring host. Entomol. Exp. Appl..

[61] Klemperer H.G. (1978). The repair of larval cells and other larval activities in *Geotrupes spiniger* Marsham and other species (Coleoptera, Scarabaeidae). Ecol. Entomol..

[62] Mallampalli N., Barbosa P., Weinges K. (1996). Effects of condensed tannins and catalpol on growth and development of *Compsilura concinnata* (Diptera: Tachinidae) reared in gypsy moth (Lepidoptera: Lymantriidae). J. Entomol. Sci..

[63] Gentry G.L., Dyer D.L. (2002). On the conditional nature of neotropical caterpillar defenses against their natural enemies. Ecology.

[64] Rogers M.E., Cole T., Ramaswamy S., Potter D.A. (2003). Behavioral changes in Japanese beetle and masked chafer grubs following parasitism by tiphiid wasps. Environ. Entomol..

[65] Rogers M.E., Potter D.A. (2002). Kairomones from scarabaeid grubs and their frass as cues in below-ground host location by the parasitoids *Tiphia vernalis* and *Tiphia pygidialis*. Entomol. Exp. Appl..

[66] Walker A.M., Hoy M.A. (2003). Responses of Lipolexis oregmae (Hymenoptera: Aphidiidae) to different instars of Toxoptera citricida (Homoptera: Aphididae). J. Econ. Entomol..

[67] Hance T., van Baaren J., Vernon P., Boivin G. (2007). Impact of temperature extremes on parasitoids in a climate change perspective. Annu. Rev. Entomol..

[68] Thomas M.B., Blanford S. (2003). Thermal biology in insect-parasite interactions. Trends Ecol. Evol..

[69] Fellowes M.D.E., Kraaijeveld A.R., Godfray H.C.J. (1999). Cross-resistance following artificial selection for increased defense against parasitoids in *Drosophila melanogaster*. Evolution.

[70] Hegazi E., Khafagi W. (2005). Developmental interaction between suboptimal instars of *Spodoptera littoralis *(Lepidoptera: Noctuidae) and its parasitoid *Microplitis rufiventris *(Hymenoptera: Braconidae). Arch. Insect Biochem. Physiol..

[71] Blumberg D. (1991). Seasonal variations in the encapsulation of eggs of the encyrtid parasitoid *Metaphycus stanleyi *by the pyriform scale, *Protopulvinaria pyriformis*. Entomol. Exp. Appl..

[72] Castelo M.K. (2012). Attachment preference of larvae of *M. ruficauda *on the tegument of white grubs. Unpublished work.

[73] Rao A., Henderson R.E., Vinson S.B. (2009). The probable significance of tracheal tufts in the 8th abdominal segment of *Heliothis virescens* (F.) on the development of its parasitoid, *Toxoneuron nigriceps* (Viereck). J. Insect Physiol..

[74] Hassell M.P. (2000). The Spatial and Temporal Dynamics of Host-Parasitoid Interactions.

[75] Comins H.N., Hassell M.P., May R.M. (1992). The spatial dynamics of host-parasitoids systems. J. Anim. Ecol..

[76] Godfray H.C.J., Hassell M.P., Holt R.D. (1994). The population dynamic consequences of phenological asynchrony between parasitoids and their hosts. J. Anim. Ecol..

